# A five-year review on death of 1-59 month children due to drowning in Hormozgan province

**Published:** 2019-07

**Authors:** Shirin Soltani, Soghra Kamali, Elham Salehi, Soheila Moradi

**Affiliations:** ^*a*^Mother and Child Research Center, Hormozgan University of Medical Sciences, Iran.

## Abstract

**Background:**

Prevention of injury and incident should be recognized as an important means in the life of children. All communities are responsible for preventing child injuries. Safe and sustainable environment is a prerequisite for a healthy community. Children should be provided with highest levels of health. They should live in a safe environment. They are close to water as soon as they begin to live. But, despite the great benefits of water for the health and survival of children, it can be dangerous. Considering that drowning in Hormozgan province is one of the major causes of death due to unintentional injuries, we decided to study the five-year mortality rate of 1- to 59-month children due to drowning in order to resolve this matter with a proper planning.

**Methods:**

This was a retrospective descriptive-analytic study performed to investigate the death of children aged 1-59 months due to drowning in Hormozgan province during 2013-2017. The data were collected by interviewing to answer to questionnaires’ items. The questionnaires were completed by a team after death in hospital (intrahospital death) or at home (extrahospital death), questioning the family of the dead child. The data were analyzed by SPSS software and t-test.

**Results:**

Over the past five years, drowning after traffic incidents has been the most common cause of death due to unintentional accidents. Of 63 deaths due to drowning, 19 cases happened in pools (30.1%), 14 in natural water resources (22.2%), 10 in the water buckets (15.9%), 7 in the agricultural water ponds (11.1%), 6 in tub (9.5%), 3 in wastewater wells (4.8%), 3 in yard's water storage (4.8%), and 1 in gasoline reservoirs at home (1.6%).

**Conclusions:**

Sensitizing families, NGOs and policymakers seems to be very effective in preventing drowning in children. Therefore, it should be considered as a health priority in the country.

**Keywords:**

Children, Mortality, Drowning

